# Brain MRI sequence and view plane identification using deep learning

**DOI:** 10.3389/fninf.2024.1373502

**Published:** 2024-04-23

**Authors:** Syed Saad Azhar Ali

**Affiliations:** Aerospace Engineering Department and Interdisciplinary Research Center for Smart Mobility and Logistics, King Fahd University of Petroleum and Minerals, Dhahran, Saudi Arabia

**Keywords:** brain MRI, sequence identification, view plane, deep learning, computer aided diagnosis, assistive tool

## Abstract

Brain magnetic resonance imaging (MRI) scans are available in a wide variety of sequences, view planes, and magnet strengths. A necessary preprocessing step for any automated diagnosis is to identify the MRI sequence, view plane, and magnet strength of the acquired image. Automatic identification of the MRI sequence can be useful in labeling massive online datasets used by data scientists in the design and development of computer aided diagnosis (CAD) tools. This paper presents a deep learning (DL) approach for brain MRI sequence and view plane identification using scans of different data types as input. A 12-class classification system is presented for commonly used MRI scans, including T1, T2-weighted, proton density (PD), fluid attenuated inversion recovery (FLAIR) sequences in axial, coronal and sagittal view planes. Multiple online publicly available datasets have been used to train the system, with multiple infrastructures. MobileNet-v2 offers an adequate performance accuracy of 99.76% with unprocessed MRI scans and a comparable accuracy with skull-stripped scans and has been deployed in a tool for public use. The tool has been tested on unseen data from online and hospital sources with a satisfactory performance accuracy of 99.84 and 86.49%, respectively.

## 1 Introduction

Neurological disorders are among the top three causes of death globally (World Health Organization [WHO], 2018). Since the past decade, medical experts and data scientists have been working in collaboration to design systems that can assist in timely and accurate diagnosis of such disorders. This has immense importance to cure or contain the progression of diseases, specially in case of neurodegenerative disorders (Miotto et al., 2018). Brain MRI is a widely used, non-invasive and informative modality when it comes to diagnosis of such disorders (Bento et al., 2022). There exists a wide variety of such scans ranging from structural to functional MRI. In addition to the “Gold Standard” analysis of brain MRI scans by neuroradiologists for differential diagnosis, this modality has been widely employed to train artificial intelligence (AI) based systems to assist medical experts as CAD tools (Tăuţan et al., 2021). In this direction, a tremendous amount of research can be seen catering single disease diagnosis, whereas only a handful of systems capable of handling CAD of multiple neurological disorders exist (Rudie et al., 2020). Extensive research can also be seen in prior art catering preprocessing of such scans. These systems include brain extraction/skull-stripping (Hoopes et al., 2022) and lesion segmentation (Hashemi et al., 2022), among others. Skull stripping can assist in the removal of extra-cranial tissue. Such artifacts including skull, neck and orbitals might hamper the disease classification performance of a CAD system which requires focus on the brain region only. In addition, operations like lesion segmentation can assist in the differential diagnosis of demyelinating diseases which result in plaque formations in the brain (Ma et al., 2022). Similar brain lesions can develop in diseases like multiple sclerosis (MS) and neuromyelitis optica (NMO), making the differential diagnosis using brain MRI alone quite tedious for medical experts (Xin et al., 2020). AI, statistical and topological analysis (Xin et al., 2020) can be helpful in such scenarios. In order to train deep networks for classification of multiple neurological disorders, a large, labeled training dataset is mandatory (Zaharchuk et al., 2018). Substantial datasets are publicly available online to help data scientists train such CAD tools. However, these datasets are generally acquired from various medical centers across the globe and given the wide set of variations available in radiological imaging, in addition to the different manufactured models of MRI acquisition hardware, there is an absolute need of sorting the MRI scans, specially based on the sequence type. This is important as a particular MRI sequence and view plane might be required while building a large cohort to train a CAD system (Helm et al., 2024). To handle such situations, some MRI file types [e.g., digital imaging and communications in medicine (DICOM), widely used in clinical practice] offer metadata pertaining to the scan in file headers (Na et al., 2023). These headers contain information like MRI sequence, body part scan, magnet strength, etc., mostly entered manually by technicians at the time of acquisition. However, the information contained in such headers might not always be consistent and accurate and even absent in some files, and is therefore not a reliable source for big data management. According to literature, approximately 16% of the information present in such headers is found to be inaccurate. In addition, to ensure anonymity in research settings, the DICOM tags are removed, rendering important information for data management unavailable (Baumgärtner et al., 2023). Other MRI data types commonly available in online sources include Neuroimaging Informatics Technology Initiative (NIfTI) volumetric data, and Jpeg/Png, etc., which do not contain such metadata. The only possible way to avoid such misclassifications from inaccurate DICOM headers information or absence of metadata, is the “Gold Standard” manual examination by radiologist, which can be a very tedious and time-consuming process given the massive data available online (Baumgärtner et al., 2023). In such scenarios, automatic identification of the MRI sequence and slice view plane from raw MRI data can prove to be of immense significance in sorting these massive datasets out for training DL models (Pizarro et al., 2019; Liang et al., 2021). This research is a humble endeavor in this direction. Multiple 12-class DL models are trained, ranging from simple 4-layer convolutional neural networks (CNNs) to more sophisticated models and transfer learning, using raw and skull-stripped MRI scans. The 12 classes include T1-axial, T1-coronal, T1-sagittal, T2-axial, T2-coronal, T2-sagittal, PD-axial, PD-coronal, PD-sagittal, FLAIR-axial, FLAIR-coronal and FLAIR-sagittal. Various publicly available datasets and resources from hospitals have been used to train and test the models. Ultimately, a MATLAB app (with the best performing model MobileNet-v2) has been developed to help data scientists manage and organize big medical imaging data for CAD research.

The organization of this paper is such that the research conducted in this direction is given in section “2 Prior art.” Section “3 Methodology and materials” provides the details of the hardware, software, datasets and algorithms used. The results and graphical user interface (GUI) design of the developed tool are presented in section “4 Results and discussion,” whereas the paper is concluded in section “5 Conclusion.”

## 2 Prior art

Medical imaging has proved to be an irreplaceable asset to experts for the diagnosis of multiple disorders. Among the various modalities available, brain MRI is widely used for diagnosis of neurological disorders by neuroradiologists. Brain MR images possess very similar features specially in case of neurodegenerative disorders (Avants et al., 2008), and some disorders in their infancy might get overlooked (Nemoto et al., 2021). Intelligent systems for identification of diseases can play a pivotal role in such scenarios (Miotto et al., 2018). The open sharing of MRI data in the neuroscience community has granted us access to big data. But with big data comes a definite need for fast and automated data organization and management. Multi-center neuroimaging databases consistently receive MRI data with unlabeled or incorrectly labeled contrast (Pizarro et al., 2019). There is a need for automated labeling of such vast data in order to save valuable resources and time spent if done manually by visual inspection. To date, very limited research in this direction has been observed while reviewing the literature. The study in Helm et al. (2024) proposes an automated method to classify chest, abdomen and pelvis MRI using DenseNet-121. Their system claims to achieve an F1 score of 99.5% at classifying 5 MRI types obtained from three Siemens scanners. Another study (Baumgärtner et al., 2023) employs 3D ResNet18 to classify 10 MRI sequence types using prostate MRI volumes from 1,243 patients. The accuracy achieved by this system is 99.88% ± 0.13%. A similar study in Lim et al. (2022) uses image based CNN for cardiac MRI sequence type and imaging plane classification. Using 434 patients from 3 centers, they achieved a classification accuracy 86.6% when tested on data not seen during training. Such studies, although not designed for human brain MRI, build the confidence in developing DL systems capable of classifying brain MRI sequences and view planes. An important aspect to observe here is the generalizability issue of such systems. Although they perform well on the test subsets of the training data, their accuracy tends to reduce when tested on unseen data from different sources.

Liang et al. (2021) explored the possibility of MRI sequence identification using metadata. They employed Random Forest and concluded that machine learning can be used to predict MRI sequence with an accuracy of 99.9%. A similar study (Na et al., 2023) proposes a self-supervised ML approach for MRI sequence type classification using DICOM metadata. They use a total of 1,787 brain MRI scans and claim to have achieved an accuracy of up to 99.7% using multi-center datasets. Such systems hugely rely on the metadata entries in the DICOM file headers, and are prone to absolute failure in case of inaccuracies in the header entries. In addition, such systems cannot be employed if MRI data is available in other file formats including NIfTI volumes (with no metadata) which is predominantly preferred in research settings.

Researchers in van der Voort et al. (2021) design a CNN to recognize 8 different brain MRI sequences including T1w, contrast enhanced T1w (T1c), T2w, PD, T2w-FLAIR, diffusion-weighted imaging (DWI) and perfusion-weighted dynamic susceptibility contrast (PWI-DSC). They use online data sources for training and testing using brain tumors and Alzheimer’s datasets and claim to achieve an accuracy of 98.5%. Data from actual clinical settings usually have different acquisition parameters including slice spatial and temporal resolutions and magnet strengths, and hence the accuracy generally drastically reduces when such systems are tested on data from hospital sources. Although the number of sequences handling capability of this system is impressive, it has no provision to cater view plane classification. The study in Pizarro et al. (2019) propose a CNN architecture to infer MRI contrast from multiple MRI slices with less than 0.2% error rate, but they also suggest that smaller datasets employed during training caused the DL system to lose generalizability. Researchers in Gao et al. (2023) develop a content-based brain MRI sorting and artifacts detection system. They use 22,092 MRI volumes from 4,076 patients containing T1w, T1c, T2w and FLAIR sequences. Although the dataset employed is reasonable, their system provides an accuracy of 99.1% which still has a room for improvement. A similar study in Ranjbar et al. (2020) employs DL to classify T1w, T1w post-gadolinium contrast (T1Gd), T2w and FLAIR MRI sequences, but use only 9,600 images of brain tumor patients for training. The accuracy claimed by this system on the test subset of the same dataset (2,400 images) is 99% but generalizability remains the most prominent limitation of this system. Similarly, the research in de Mello et al. (2020) proposes ResNet architecture to classify volumetric brain scans as FLAIR, T1w, T1c or T2w. They used publicly available datasets (BraTS and TCGA-GBM) to train the system producing a test accuracy of 96.81%. The performance of this system using data from clinical sources or other online datasets not incorporated during training has not been evaluated and is deemed to degrade due to poor generalizability of such systems. No work has been found to cater slice view plane identification. ResNet-50 has been found to produce popular results for classification systems with brain MRI as input. Multiple researches were found in literature using transfer learning with ResNet-50 architecture for various multiclass classification applications ranging from brain tumor detection (Divya et al., 2020) to diagnosis of Alzheimer’s disease (Fulton et al., 2019). It has therefore been included in the experiments conducted in this research along with other architectures. In addition, MobileNet-v2 can be an interesting choice given its lightweight architecture which enables deployment in mobile devices with limited computational resources.

The datasets and methods used in the development of MRI sequence and view plane identification system are given in the subsequent section.

## 3 Methodology and materials

**FIGURE 1:**
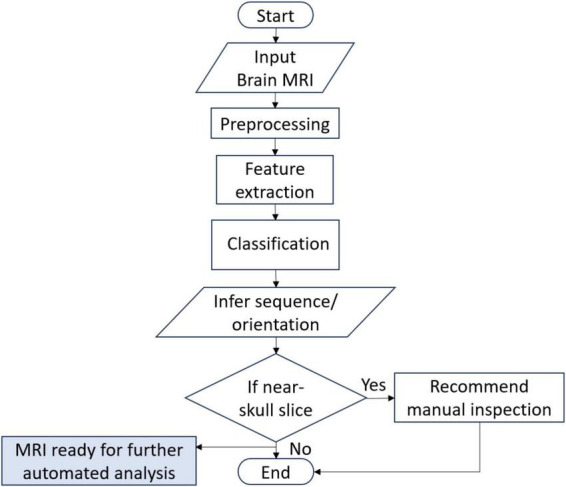
System flowchart.

The flow of the system developed in this research is given in Figure 1. The system can accept brain MRI scans in multiple file formats including JPEG, PNG, BMP and DICOM. Once the input is provided, it is preprocessed to match the input requirements and passed to the DL model. The DL model extracts features and infers the sequence and view plane of the MRI scan. In addition, the relative position of the brain slice is also identified using non-zero pixel elements in the image and thresholding. If the slice is identified to be a near skull (not mid-brain) slice, it is recommended to manually verify the inference by visual inspection by an expert, since it is quite difficult to distinguish between multiple MRI sequences and view planes if there is very little brain content in the scan. The details of the datasets used for training, validation and testing, the DL architectures employed in this research, the hardware used for training and development, along with the graphical user interface (GUI) are also provided in this section.

Magnetic resonance imaging (MRI) scans from three publicly available datasets, the neurofeedback skull-stripped (NFBS) Repository (Puccio et al., 2016), the SynthStrip dataset (Hoopes et al., 2022) and MICCAI 2016 Challenge Dataset (MSSEG) (Commowick et al., 2021b) were used for training, validation and testing. T1, T2-weighted, FLAIR and PD MRI sequences in axial, coronal and sagittal view planes were used in this study. The number of slices extracted from the publicly available datasets for different sequences and view planes are given in Table 1. MR scans are available as NIfTI volumes in these datasets and individual slices were extracted for training.

**TABLE 1:** Images acquired from MRI scans in three publicly available datasets.

	Axial	Coronal	Sagittal
**NFBS dataset**			
T1	2,000	2,000	2,000
**SynthStrip dataset**			
T1	16,840	18,532	16,645
T2	11,071	11,125	8,593
FLAIR	389	0	0
PD	10,210	10,414	8,057
**MSSEG 2016 dataset**			
FLAIR	11,302	15,205	7,576

Table 2 presents the dataset summary with the total images extracted in each category to be used for training, validation and testing. A 90/10/10% train, validate and test split was used for training. It can be seen from Table 2 that FLAIR sequences in sagittal view plane have the least number of images (7,576) and hence 750 images (10%) from each class were separated for validation and testing. The MR images were then rotated by 90, 180, and 270° before training making a total 535,823, 36,000 and 36,000 images used for training, validation and testing, respectively.

**TABLE 2:** Summary of the dataset used for training, validation and testing.

	Axial	Coronal	Sagittal
T1	18,840	20,532	18,645
T2	11,071	11,125	8,593
FLAIR	11,691	15,204	7,576
PD	10,210	10,414	8,057

In addition, 3 subjects were taken for testing from different publicly available datasets [IXI (Imperial College London, 2022), MICCAI 2021 (MSSEG-2) (Commowick et al., 2021a) and ADNI (Bernick, 2022)] which were not used in training. The details of subjects are given in Table 3. Also, an additional 3 cases acquired from Advanced International Hospital (AIH) Islamabad were used for testing, and the scans and view planes used for subjects 1, 2 and 3 were T1 axial, T2 sagittal and FLAIR coronal, respectively. The summary of these cases is given in Table 4.

**TABLE 3:** Test subjects from online datasets.

Dataset	Subject	File	Sequence	View plane	Slices
IXI	IXI-002-Guys-0828-PD	NIfTI	PD	Axial	86
				Coronal	131
				Sagittal	125
IXI	IXI-002-Guys-0828-T2	NIfTI	T2	Axial	86
				Coronal	131
				Sagittal	126
MSSEG-2 (2021)	2a_isovox “Unsupervised folder”	NIfTI	FLAIR	Axial	106
				Coronal	96
				Sagittal	101
ADNI 1.5 Tesla	002_S_0413	NIfTI	T1	Axial	71
				Coronal	131
				Sagittal	86

**TABLE 4:** Test subjects from Advanced International Hospital (AIH) Islamabad dataset.

Subject	Gender	Age	File	View plane	Sequence	Slices
1	Male	27	DICOM	Axial	T1W	19
**Clinical information:** Diagnosed case of HIV. Presented with fits, desaturation and hypotension						
2	Female	26.2	DICOM	Sagittal	T2W	20
**Clinical information:** Seizure disorder						
3	Male	36.1	DICOM	Coronal	FLAIR	35
**Clinical information:** Fits						

ResNet-50, AlexNet, MobileNet-v2 and two additional 4- and 7-layer CNNs were chosen to train 12-class classification models with T1 axial, T1 coronal, T1 sagittal, T2 axial, T2 coronal, T2 sagittal, PD axial, PD coronal, PD sagittal, FLAIR axial, FLAIR coronal and FLAIR sagittal as the classes. The learnable parameters of the afore mentioned architectures were found to be ranging from 1.5 to 60.9 M. NeuroImaging volumetric extractor (NIVE) (Khuhed, 2023) was used for skull stripping. Both grayscale and 3-channel images were used as input to the models with a resolution of 256 × 256 and 224 × 224.

**FIGURE 2:**
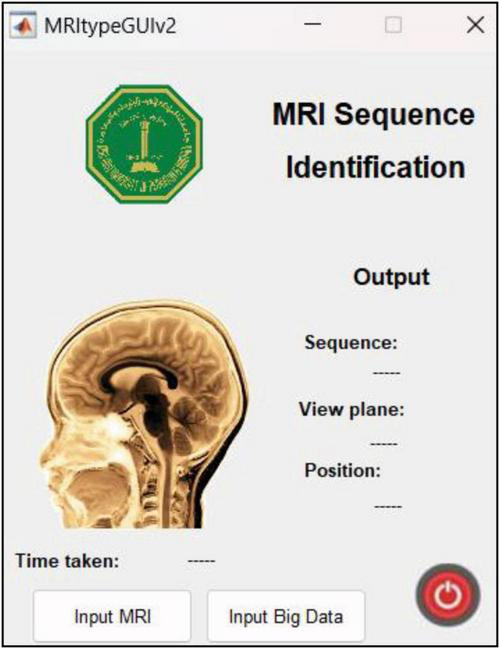
UI designed in MATLAB. The “input MRI” pushbutton accepts MRI file in a dialog box. The “input big data” pushbutton requests for a path to multiple MRI scans and classifies them, thereby reducing time and requiring minimum human intervention. The sequence, view plane and relative slice position are displayed along with the inference time.

This research was carried out using Intel^®^ Core™ i7-9750H CPU @ 2.60GHz 2.59GHz Lenovo Legion Y545 with 16GB RAM and 6GB NVIDIA GeForce GTX 1660 Ti. Another Linux based machine with 12GB NVIDIA GeForce RTX 2080 Ti was also used for training with MATLAB r2022b. MATLAB graphical user interface development environment (GUIDE) was used to design the user interface (UI) of the tool with the deployed DL model. The UI is shown in Figure 2. The results of the MRI sequence and view plane identification tool are presented in the following section.

## 4 Results and discussion

This section mainly presents the results of the model (MobileNet-v2) with optimum performance accuracy and speed, among the various models trained in this research. A comparative analysis and the journey taken to MobileNet-v2 is presented at the end of this section (section “4.4 Discussion”). The first model was trained with MRI scans without removing the extra-cranial tissue. This model was trained for 4 epochs with a learning rate of 0.01. Further details are given in section “4.1 Performance evaluation with original (with-skull) MRI scans.” The second model was trained with the same learning rate for 4 epochs using the skull-stripped versions of the same MRI dataset. NIVE was used to carry out brain extraction automatically. In this case, however, some of near-skull MRI slices were discarded before training the model, since such images contained very little or no brain content for the DL model to make sense of and hence were expected to degrade the performance. The details are given in section “4.3 Performance evaluation with skull-stripped MRI scans.” The performance comparison with and without using ImageNet weights during training is given in section “4.2 MobileNet-v2 performance comparison with and without pre-trained weights.” The app user interface and features are given in section “4.5 App user interface and features” along with the inference speed comparison.

### 4.1 Performance evaluation with original (with-skull) MRI scans

**FIGURE 3:**
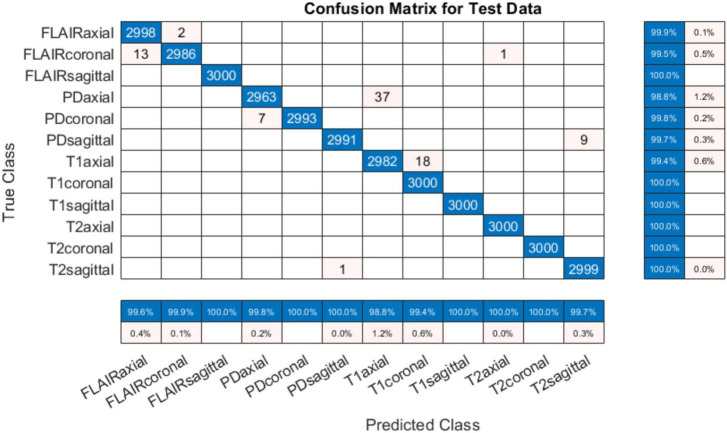
Confusion matrix for 12-class classification system (with skull).

**FIGURE 4:**
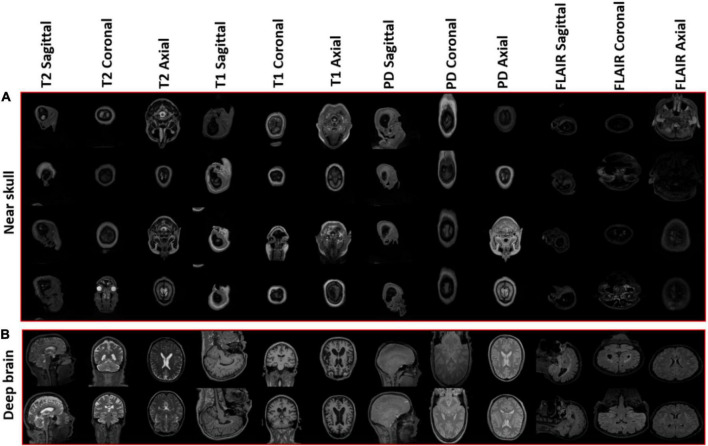
**(A)** Near skull images with high probability of invalid inferences **(B)** deep brain images resulting in optimum performance.

The training progress of MobileNet-v2 is given in section “4.2 MobileNet-v2 performance comparison with and without pre-trained weights.” The validation and testing accuracies were evaluated to be 98.28 and 99.76%, respectively. The confusion matrix of the developed model on 10% testing data split from the same publicly available datasets used in training is shown in Figure 3. From Figure 3, it can be observed that some classes have been predicted flawlessly, nonetheless some inaccuracies can still be seen. 37 PD axial images have been misclassified as T1 axial, and some PD sagittal have been misclassified as T2 sagittal. It can be noticed here that at least, as expected, the view plane has been inferred correctly by the DL model in these cases. Further investigation suggested that the presence of near skull slices (with very little cranial tissue) was the cause of these inaccuracies. Since it is very difficult to judge the sequence and view plane of a near skull brain MRI slice even by visual inspection. The system performs perfectly for deep brain slices. Figure 4 shows the near skull and deep brain slices for all sequences and view planes. Since the extracranial tissues do not help much in the diagnosis of neurological disorders and the brain content in such edge slices is either zero or very less, these slices can be discarded. Traditionally, this is achieved using skull stripping/brain extraction tools like SynthStrip (Hoopes et al., 2022) and NIVE. Discarding only 0.5% of these near skull images resulted in an improvement in the validation and test accuracy. The trained model was then deployed in a tool/app in MATLAB. To cater the prediction inaccuracies in near skull images, the app has a feature to distinguish between near skull and deep brain slices. The inference for near skull images can therefore be cross-examined manually by experts to rule out any misclassifications by the system.

#### 4.1.1 Testing on unseen online data

**FIGURE 5:**
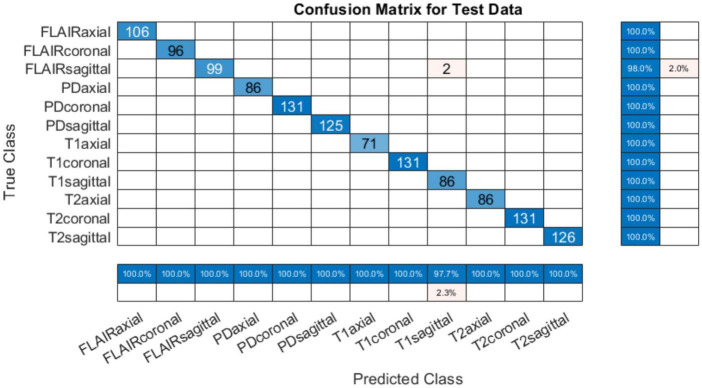
Confusion matrix on test data from IXI, MICCAI 2021 and ADNI datasets (with skull).

The developed system was tested on unseen data available online from different sources are detailed in Table 3. Four subjects taken from IXI, MICCAI 2021 and ADNI in all view planes were used during this test. The results obtained from testing the developed system were quite promising with an accuracy of 99.84%. The confusion matrix is given in Figure 5. It can be observed that out of 1,276 images, only 2 were incorrectly classified. It is worth mentioning that the sagittal view planes of these two images were correctly classified nevertheless, despite the confusion between FLAIR and T1 sequences.

#### 4.1.2 Testing on AIH Islamabad data

**FIGURE 6:**
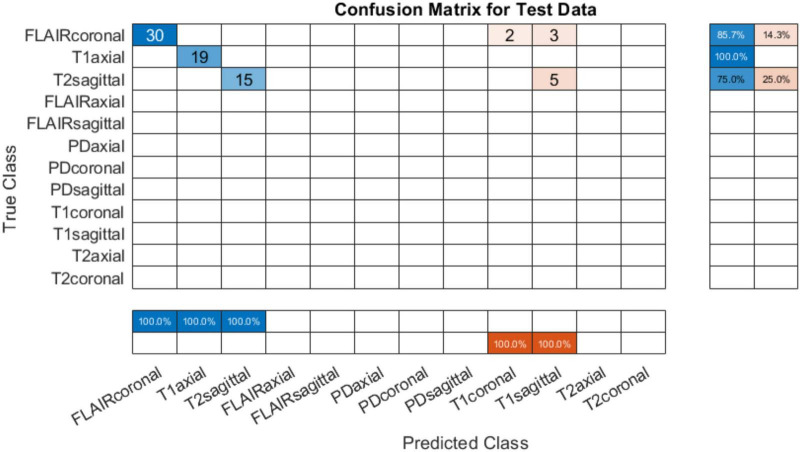
Confusion matrix on test data from AIH Islamabad (with skull).

The developed system was tested on data from AIH Islamabad as detailed in Table 4. Results showed that T1 axial images were perfectly classified. However, the FLAIR coronals can be seen misinterpreted as T1 coronals and T2 sagittals being mixed up with T1 sagittals. Figure 6 shows the confusion matrix for classification using AIH data. The performance accuracy was evaluated to be 86.49%. The performance of the developed system on AIH data was further investigated and it was observed that the data has low spatiotemporal resolution which may be the reason for low accuracy. Therefore, to verify the generalizability of the developed tool, an additional dataset was acquired from Pakistan Institute of Medical Sciences (PIMS) Pakistan. The details are given in the subsequent section.

#### 4.1.3 Testing on PIMS data

**FIGURE 7:**
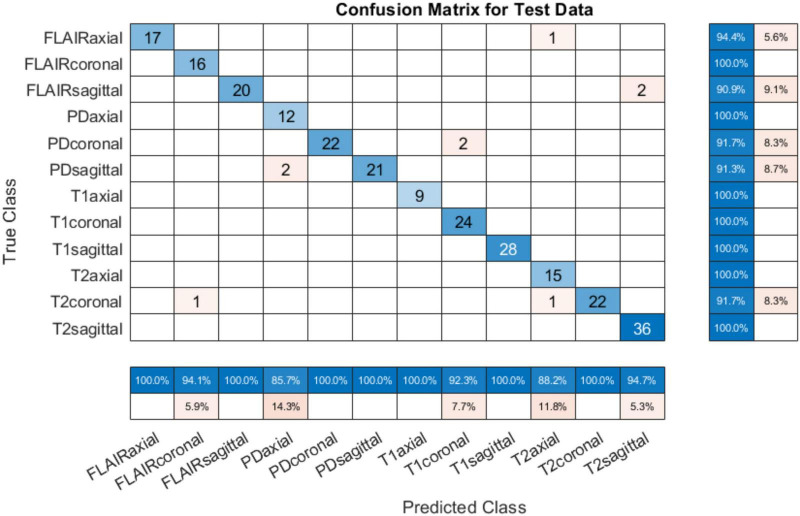
Confusion matrix on PIMS additional test data.

To test the developed system for all classes, another set of test data was requested from PIMS hospital with one subject belonging to each of the twelve classes. The test subjects had different pathologies with a higher spatiotemporal resolution. The number of slices per sequence is given in Table 5. The developed tool tested on PIMS data resulted in a reasonably higher accuracy of 96.41% compared to AIH data. The confusion matrix is given in Figure 7. Testing on a varied data builds confidence in the generalizability of the developed system.

**TABLE 5:** 12 cases obtained for PIMS for further testing.

Sequence	T1	T2	FLAIR	PD
Slices	61	75	56	59
Total	251			

It can be observed from the results in Figure 7 that almost all sequences and slice view planes have been correctly classified. On further analysis of the confusion matrix, it was interesting to observe that although some of the MRI sequences were misclassified, the view plane were still correctly classified. For example, two PD coronal slices were classified as T1 coronal slices. Similarly, two FLAIR sagittal slices were classified as T2 sagittal slices. This poses a challenge to the classification algorithm where the FLAIR sequence is very similar the T2 sequence,

other than the normal cerebrospinal fluid (CSF) attenuation. In addition, it can be quite challenging to infer the sequence and slice view plane in case of near-skull images, with very little brain content. This nevertheless is a concern which can be addressed by increasing training dataset and adding more MRI scans from different sources/hospitals, and acquired from different hardware. This will result in better generalizability of the trained model. The performance of the system on the data from three online sources not used during training, and from consultant radiologists in clinical settings at two different institutions builds our confidence in the generalizability of the system.

### 4.2 MobileNet-v2 performance comparison with and without pre-trained weights

**FIGURE 8:**
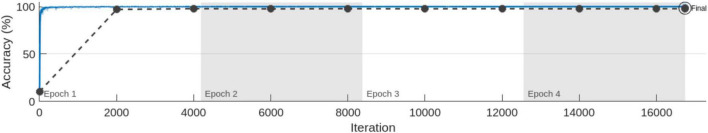
MobileNet-v2 Training for 4 Epochs (with skull) using transfer learning.

**FIGURE 9:**
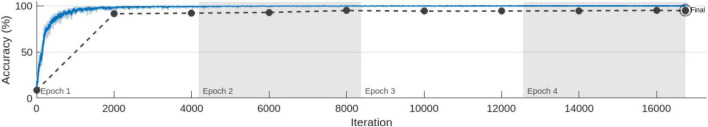
MobileNet-v2 Training for 4 Epochs (with skull) without using pre-trained weights.

In the series of experiments, MobileNet-v2 architectures were trained with and without using ImageNet pre-trained weights for transfer learning. The training processes are shown in Figures 8, 9, respectively. It can be observed that the model using pre-trained weights has learnt faster with a higher accuracy. Multiple test accuracies for both models are given in Table 6 for comparison.

**TABLE 6:** Comparison of testing accuracies with and without the use of ImageNet weights.

Accuracy	MobileNet-v2 (without using weights)	MobileNet-v2 (with weights)
Validation	95.27%	98.28%
Test	97.51%	99.76%
Online test	83.93%	99.84%
AIH test	64.86%	86.49%

### 4.3 Performance evaluation with skull-stripped MRI scans

After extracting brain from the entire dataset (training, validation, and testing), non-zero pixels were counted in each image. Any image with a total of less than 5,000 non-zero pixels were discarded from the process. This is because the (near-skull) MRI slices with very little brain content are difficult to categorize in terms of sequence and view plane. Table 7 shows the total images discarded using the criteria mentioned above. The dataset was augmented with rotated images in the same way as done with un-preprocessed (with skull) MR images. All the models used in this research were retrained with the skull-stripped version of the dataset and produced comparable results with their (with skull) counterparts. Numeric values of accuracies have not been provided here since some near skull MR slices were discarded while training this model, hence a statistical comparison is not possible here. Since no substantial leaps in accuracy were observed, the developed app has been embedded with the MobileNet-v2 model trained using the un-preprocessed version of the dataset. The skull-stripping experiment was fruitful in establishing that sequence and view plane classification is also possible for skull-stripped MRI data.

**TABLE 7:** Near skull images excluded from training, validation and testing dataset.

Sequence	View plane	Train	Validation	Test
FLAIR	Axial	1,507	82	117
	Coronal	2,151	92	99
	Sagittal	985	119	129
PD	Axial	1,682	144	127
	Coronal	1,406	73	122
	Sagittal	739	99	106
T1	Axial	2,678	129	136
	Coronal	3,092	150	146
	Sagittal	2,132	84	90
T2	Axial	1,799	123	127
	Coronal	1,479	127	132
	Sagittal	818	195	98
Total deleted		20,468	1,327	1,429

### 4.4 Discussion

This section sheds light on the journey taken through alternate workflows and the motivation for using MobileNet-v2. The task of classifying different MRI view planes seems pretty straight forward for DL to handle because of the absolutely vivid differences between axial, coronal and sagittal view planes. These differences are fairly simple to identify even for people not familiar with the MRI technology and scans, and can be correlated to being as simple as distinguishing a cat from a tree. Distinguishing between MRI sequences (like T1 and T2-weighted) on the other hand might be slightly tricky for people not related to the field. Nevertheless, this task is extremely easy when compared to distinguishing between MRI scans of an Alzheimer’s and a Parkinson’s disease patient. These neurodegenerative disorders do not produce plaques, lesions or instantly visible scars on the brain like those seen in cases of multiple sclerosis or brain tumors. Keeping this context in mind, a simple 4-layer CNN was designed consisting of 3 convolutional layers and a fully connected layer to carry out this job. Fewer convolutional layers would just look at the bigger picture and ignore the more intricate and subtle features which in this case were not required. This would result in a reduction of learnable parameters and computational times. Grayscale input images (256 × 256 resolution) were used to train this network. A learning rate of 0.01 was maintained throughout the experiments. The results after 4 epochs of training were not convincing, with a validation and test accuracy of 78.29 and 77.19%, respectively. The accuracy of this model on AIH dataset was 2.70% which led to the addition of 3 more convolutional layers in the next experiment. Although the validation and test accuracies rose to 91.71 and 93.61%, respectively, the AIH data was still not being classified reasonably (accuracy 12.16%). No further hyper-parameter tuning and experimentation was carried out in this direction, and hence, transfer learning and the existing architectures including AlexNet, ResNet50 and MobileNet-v2 were explored.

Structural brain MR images appear to be grayscale images and do not contain color information. Therefore using 3 channels in an MRI image seems redundant and a computational overhead for a DL regimen. Keeping that in mind, AlexNet and ResNet50 were modified to accept a single channel input rather than the default 3 channel input. The number of output classes was also changed to 12. They produced comparable results for validation and test accuracies ranging from 94.77 to 99.78% with multiple trainings and various changes in training data. They were separately trained using both with-skull and skull-stripped input, and retrained after eliminating 4–6% near-skull images (which are harder to classify because of very little information). The unfortunate part was their performance on AIH data which produced an accuracy ranging from 17.57 to 28.38%. Additional models were trained with and without zero-centered normalization producing no difference on accuracy.

A breakthrough in accuracy of classifying AIH data was seen after the addition of 90, 180, and 270° rotated MR images in the training dataset using ResNet50. The accuracy increased drastically in both with-skull and skull-stripped models. With the hope of further increasing the performance, more training data augmentations were introduced. A ± 10% scaling, a ± 30 pixels translation and an x/y flip were introduced during training but resulted in very slight accuracy improvement.

Finally, MobileNet-v2, a CNN architecture that seeks to perform well on mobile devices, with its 3.5 M learnable parameters, was chosen since its TFLite version can be embedded in android apps to produce fast and accurate inferences. It was trained with the dataset and training configurations that produced the best results from the previous experiments with and without using the pre-trained weights. The model trained using transfer learning learnt faster and produced an accuracy enhancement of 3.01, 2.25, 15.91 and 21.63%, respectively for validation, test, test on online dataset (not used in training), and AIH Islamabad dataset. The premise of not using the pre-trained weights and training the architecture from scratch was that the 1,000 classes of ImageNet do not contain any class related to Brain MRI. But despite that, the pre-trained weights were found to be helpful in this 12 class MRI classification application.

The developed system performed almost flawlessly, with an accuracy of 99.84%, on testing data acquired from online sources not used in training. Nevertheless, in order to ensure the reliability of the developed system for practical use, it was mandatory to test it on data acquired from hospitals. Data (labeled by consultant radiologists) from two hospitals (AIH and PIMS) was acquired for this purpose and the corresponding test accuracies were found to be 86.49 and 96.41%, respectively. It was also observed that the misclassifications that resulted in the accuracy drop were partially correct. The reason being that despite the incorrect classification of MRI sequence in some cases, yet the slice view plane was correctly classified. The explanation for incorrect sequence classification is that specially in the near-skull slices, containing very little brain content, it is difficult to distinguish between similar sequences like T2-weighted and FLAIR. To cater for this issue in the near-skull slices, a feature was added in the system to identify such slices and warn for an additional manual inspection by experts. In addition, one of the possible reasons for the lower accuracy on AIH data as compared to PIMS data could be the lower spatiotemporal resolution of the scans obtained from AIH. The overall satisfactory performance of the developed algorithm led to its deployment into an app for public use. The user interface (UI) and features of the app are given in the subsequent section.

The limitations of the developed system include the deployment in MATLAB which is a licensed software and might not be freely accessible to scientists and medical professionals. Python and Android versions of the app have also been developed and will be released publicly after further testing and peer reviews. In addition, currently the system is capable of handling only 4 commonly employed MRI sequences (T1w, T2w, FLAIR and PD) which can be extended to classify other sequences also including Diffusion-weighted imaging (DWI), perfusion-weighted imaging (PWI), susceptibility weighted imaging (SWI) and short tau inversion recovery (STIR), among others.

### 4.5 App user interface and features

**FIGURE 10:**
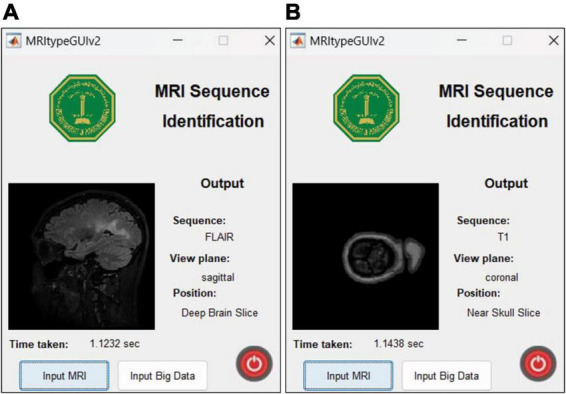
MRI Sequence Identification UI. Correct classification of **(A)** deep brain FLAIR axial slice **(B)** near skull T1 coronal slice.

The UI is extremely user friendly with a push button to input MRI. The MRI file types accepted by the tool include JPG, PNG, BMP and DICOM. Digital imaging and communications in medicine (DICOM) files are generally preferred by radiologists/neurologists and are accessed using specialized software like RADIANT DICOM Viewer (Medixant, 2023). After receiving the MRI file from user, the app displays the MRI image along with information including sequence, view plane, deep brain/near skull image and time taken to process the image. A sample of the app’s performance is shown in Figure 10. Notice that in Figure 10A, the MRI is deep brain slice as opposed to near skull slice correctly identified in Figure 10B. Also notice that the time taken per slice analysis is around 1.15 seconds when tested on a machine with 6GB NVIDIA GeForce GTX 1660 Ti. This time was up to 1.9 seconds when tested using ResNet50. This is version 1.0 of the app which can identify MRI sequences and view planes. The next version of this app will include further models for detailed analysis (Version 2.0 of this app has a provision to handle big data, with the addition of a pushbutton “Input Big Data,” as shown in Figure 2. This provision allows users to input path to a folder with multiple MRI files which can be collectively sorted without human intervention, making it a fast and easy process).

## 5 Conclusion

This research presents a system for classification of multiple brain MRI sequences and slice view planes. MRI sequence and view plane identification using MobileNet-v2 is a fast and accurate publicly available tool. It can prove to be very useful specially to data scientists and medical experts working on CAD systems for neurological disease diagnosis using brain MRI. The developed app can assist experts in automatically, quickly, and accurately labeling massive online MRI datasets for various diseases (neurodegenerative and otherwise), which would be an extremely tedious job if done manually. These labeled scans can then be used to train pipelines of CAD models for better performance. This can result in accurate and timely diagnoses saving precious lives.

## Data availability statement

The original contributions presented in this study are included in this article/supplementary material, further inquiries can be directed to the corresponding author.

## Author contributions

SA: Conceptualization, Data curation, Formal analysis, Funding acquisition, Investigation, Methodology, Project administration, Resources, Software, Supervision, Validation, Visualization, Writing – original draft, Writing – review & editing.
